# Optimal post-gastroscopy timing of the urea [^14^C] breath test for *Helicobacter pylori* diagnosis: a histopathology-anchored diagnostic accuracy study

**DOI:** 10.3389/fmed.2026.1935940

**Published:** 2026-08-25

**Authors:** Mei Wu, Xinyuan Wang, Lijuan Wang, Yining Xiang, Liangbi Xu

**Affiliations:** 1 School of Clinical Medicine, Guizhou Medical University, Guiyang, Guizhou, China; 2 Department of Gastroenterology (Endoscopy Center), Zhejiang Provincial People's Hospital Bijie Hospital, Bijie, Guizhou, China; 3 Department of Pathology, Zhejiang Provincial People's Hospital Bijie Hospital, Bijie, Guizhou, China; 4 Endoscopy Center, Affiliated Hospital of Guizhou Medical University, Guiyang, Guizhou, China; 5 Guizhou Provincial Key Laboratory for Digestive System Diseases, Affiliated Hospital of Guizhou Medical University, Guiyang, Guizhou, China; 6 Department of Pathology, Affiliated Hospital of Guizhou Medical University, Guiyang, Guizhou, China

**Keywords:** diagnostic accuracy, gastroscopy, helicobacter pylori, kyoto classification, urea [^14^C] breath test

## Abstract

**Background:**

*Helicobacter pylori* is a major cause of gastric carcinogenesis, and accurate detection of active infection guides eradication therapy and cancer prevention. The urea [^14^C] breath test (UBT) is widely used, but its reliability and optimal timing when performed on the same day after gastroscopy are uncertain.

**Methods:**

In this single-center retrospective diagnostic-accuracy study, 298 adults underwent gastroscopy followed by UBT at 1 hour (*n* = 99), 2 hours (*n* = 99), or 4 hours (*n* = 100) between December 2023 and January 2025. The reference standard was defined independently of the UBT as positive immunohistochemistry, or concordant rapid urease test and methylene blue staining in immunohistochemistry-negative cases. Agreement and diagnostic-performance measures were estimated, and histopathological characteristics of false-negative results were examined.

**Results:**

The reference-standard prevalence of *H. pylori* infection was 47.0% (140/298). Agreement was substantial to almost perfect at 1, 2, and 4 hours (κ = 0.758, 0.817, and 0.704, respectively; all *P* < 0.001). The 2-hour interval had the highest specificity (96.2%) and positive predictive value (95.2%), whereas the 4-hour interval had the highest sensitivity (95.6%) and negative predictive value (95.5%). All five false-negative results at 1 hour occurred in histopathological Stage III (*P* = 0.044). The examined procedural and mucosal-background factors were not significantly associated with UBT accuracy.

**Conclusion:**

Same-day post-gastroscopy UBT was clinically informative in this cohort. Among the evaluated intervals, 2 hours favored diagnostic confirmation, whereas 4 hours favored sensitivity-oriented screening.

## Introduction

*Helicobacter pylori* (*H. pylori*) is a World Health Organization Class I carcinogen and is responsible for a large proportion of non-cardia gastric cancers. Infection remains common worldwide and in China, and timely identification of active infection is essential for eradication therapy and gastric cancer prevention (1–5).

Diagnostic options include invasive biopsy-based tests, such as culture, histology, and the rapid urease test (RUT), and non-invasive tests, including stool antigen testing, serology, and carbon-labeled urea breath testing. The UBT has high diagnostic accuracy and is recommended as a preferred test for active infection, although medications, gastrointestinal bleeding, altered gastric anatomy, dietary conditions, and non-*H. pylori* urease-producing organisms may affect results (6–9). The RUT provides rapid results during endoscopy but can be false negative when bacterial density is low; appropriate multi-site biopsy sampling improves diagnostic yield (10–13).

The urea [^1^^3^C] and urea [^14^C] breath tests share the same biological principle: *H. pylori* urease hydrolyzes labeled urea, and labeled carbon dioxide is measured in expired breath. The [^1^^3^C] test uses a stable, non-radioactive isotope and commonly requires isotope-ratio mass spectrometry or infrared spectrometry, whereas the [^14^C] test uses a very low-dose radioactive tracer detected by a dedicated counter. Both can provide high diagnostic accuracy, but their analytical platforms, reporting units, cut-off values, and patient-use considerations are not interchangeable (6, 7). The present study specifically evaluates the capsule-based urea [^14^C] breath test in adults.

Gastroscopy is frequently completed before UBT in same-day clinical pathways because fluid ingested for breath testing can impair mucosal visualization. White-light endoscopy remains widely accessible, and the Kyoto classification provides standardized features associated with current, previous, or absent *H. pylori* infection. Advanced imaging can improve characterization, but availability and performance vary across settings (14–24). Whether gastric instrumentation, irrigation, biopsy, and mucosal preparation alter the accuracy of a subsequently performed UBT remains insufficiently defined.

Histopathology provides an independent tissue-based anchor for evaluating UBT performance. The updated Sydney system grades *H. pylori* density and gastritis, and immunohistochemistry is particularly useful when bacterial density is low or morphology is atypical (25–35). Linking UBT discordance to histopathological stage may therefore help explain false-negative results and identify mucosal contexts in which post-gastroscopy testing is less reliable.

Same-day testing may reduce repeat visits and accelerate treatment decisions, but the interval must also allow recovery from sedation and meet fasting requirements. Post-endoscopy monitoring commonly extends for at least 30 min, swallowing function generally recovers within approximately 1 h, and oral intake is often resumed after 2 h (36, 37). The 1-, 2-, and 4-h intervals evaluated here corresponded to the unit's routine scheduling windows.

Accordingly, the objectives of this study were to: (1) determine the diagnostic reliability of the urea [^14^C] breath test when performed after gastroscopy; (2) compare its diagnostic-performance profile at 1, 2, and 4 h to identify the most clinically appropriate timing for confirmation-oriented and sensitivity-oriented use; and (3) characterize the histopathological features associated with true-positive and false-negative UBT results among patients with confirmed *H. pylori* infection.

## Materials and methods

### Study design and setting

This was a single-center retrospective diagnostic-accuracy study conducted at Zhejiang Provincial People's Hospital Bijie Hospital, Guizhou Province, China, using records from December 2023 to January 2025. The study evaluated urea [^14^C] breath testing at defined intervals after gastroscopy against an independent histopathology-based reference standard and was reported in accordance with STARD 2015 (38). The protocol was approved by the hospital's Scientific Ethics Committee (Ethical Approval Number: 2023-11-1), which waived written informed consent because only de-identified retrospective records were analyzed. The study was conducted in accordance with the Declaration of Helsinki (2013 revision, the version in force when the protocol was approved) (39).

### Participants

#### Case identification

All consecutive patients who underwent elective gastroscopy followed by same-day UBT during the case-finding period were screened through the electronic medical record and day-procedure UBT registry. Because the study used an existing clinical cohort, the sample size was not fixed by a prospective power calculation; all eligible consecutive records within the predefined period were included to reduce selection bias. For transparency, we performed a retrospective precision benchmark using Buderer's method for diagnostic-accuracy studies (40). Assuming an expected sensitivity and specificity of 0.90, an infection prevalence of 0.47, a two-sided α of 0.05, and a desired 95% confidence-interval half-width of 0.10, the minimum total sample required per timing group was 74 for sensitivity and 66 for specificity. The available group sizes of 99, 99, and 100 exceeded both benchmarks. This calculation is presented as an assessment of achieved precision and does not convert the retrospective study into a prospectively powered comparative or equivalence trial.

#### Group definition

**Figure 1:**
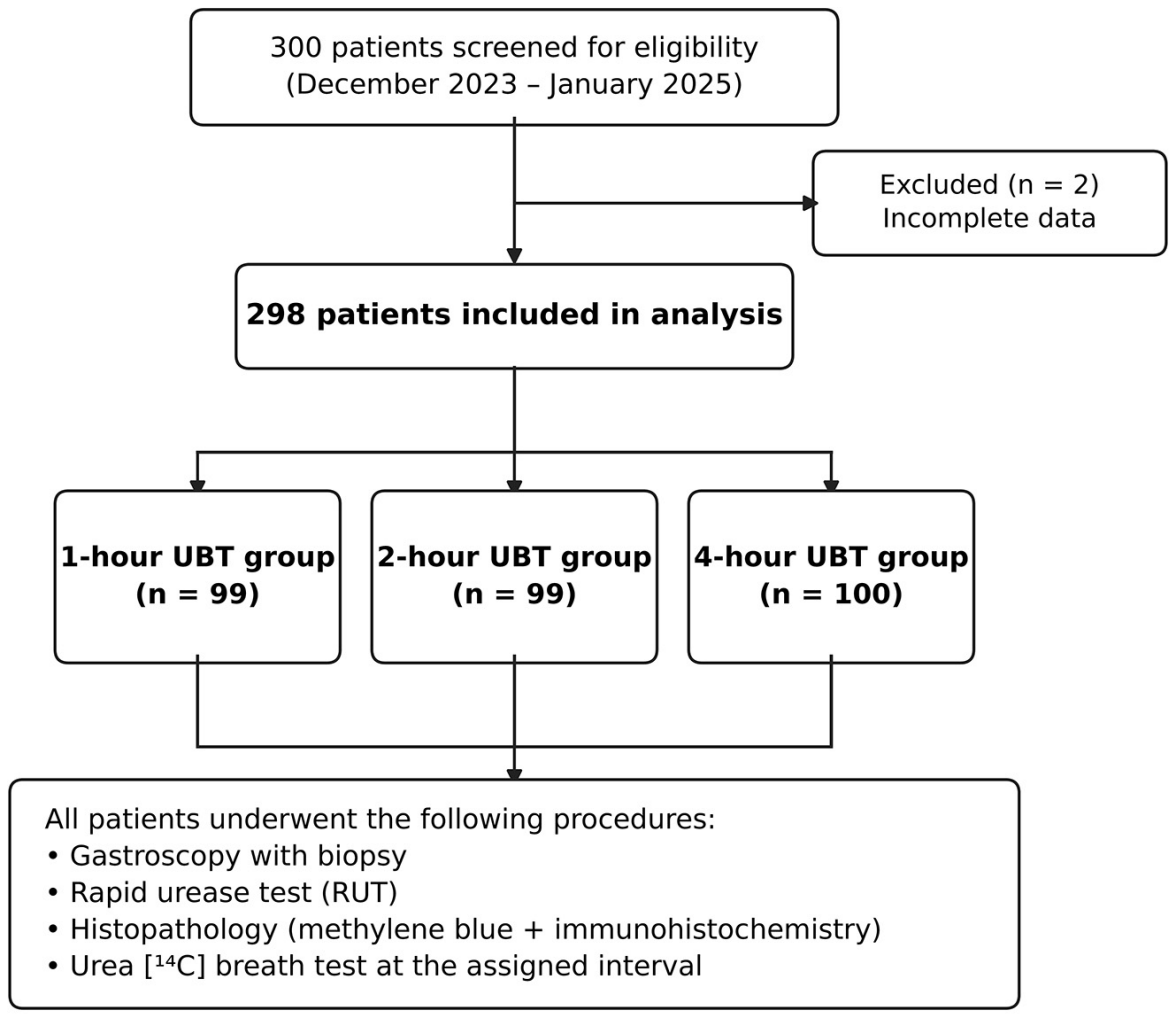
Flowchart of patient enrollment, grouping, and *H. pylori* testing procedures. From December 2023 to January 2025, 300 patients undergoing gastroscopy were screened; 2 patients were excluded because of incomplete data, leaving 298 valid cases. Patients underwent gastroscopy with biopsy, rapid urease testing, histopathological examination including methylene blue staining and immunohistochemistry, and urea [^14^C] breath testing at 1, 2, or 4 h after gastroscopy.

Patients were grouped on the basis of the actual post-gastroscopy interval at which their UBT was performed: 1 h, 2 h, or 4 h after completion of the endoscopic procedure. These three intervals corresponded to the day-procedure unit's standard UBT scheduling slots and were determined by appointment availability at the time of presentation rather than by clinical characteristics of the patient or by any researcher decision. Baseline characteristics were extracted from the electronic medical records and compared across groups to assess comparability and to identify any potential confounding from scheduling-related patient selection (Table 1). The case-identification process and patient disposition are summarized in Figure 1.

**Table 1:** Baseline characteristics of patients by post-gastroscopy time group.

General Information	Category	1 h (*n* = 99)	2 h (*n* = 99)	4 h (*n* = 100)	*P*
Sex	Male	58	52	50	0.0
	Female	41	47	50	
Place of residence	Urban	48	41	41	0.0
	Rural	51	58	59	
Ethnicity	Han	87	84	88	0.0
	Ethnic minority	12	15	12	
Smoking history	Yes	46	43	40	0.0
	No	53	56	60	
Drinking history	Yes	54	48	44	0.0
	No	45	51	56	
Food preference	Spicy diet	35	34	33	0.0
	Bland diet	64	65	67	
Age group	< 40	13	18	21	>0.05
	≥40	86	81	79	

#### Eligibility criteria

**Inclusion criteria**. Eligible patients were adults aged ≥18 years of either sex who presented with symptoms of a digestive disorder (including upper abdominal discomfort, gastroesophageal reflux, or suspected peptic ulcer disease) and had a clinical indication for diagnostic gastroscopy. Additional requirements were documented gastroscopy with biopsy for pathological examination, availability of complete and accessible clinical records, and a documented same-day UBT performed at one of the three scheduling intervals (1, 2, or 4 h after the procedure).

**Exclusion criteria**. Patients were excluded if they had any of the following: known or suspected gastric malignancy; prior upper digestive tract surgery; documented use of proton pump inhibitors, H_2_ receptor antagonists, gastric mucosal protectants, non-steroidal anti-inflammatory drugs, anticoagulants, probiotics, bismuth-containing agents, antibiotics, or herbal preparations with antibacterial activity within 2 weeks before gastroscopy; lactation; active upper gastrointestinal bleeding; hematological disorders, including anemia and bleeding diathesis; severe hepatic, renal, cardiac, or pulmonary dysfunction; or pyloric obstruction.

Of the 300 patients identified during the case-finding period, 2 were excluded after detailed record review (1 for incomplete data and 1 for an undocumented history of upper gastrointestinal bleeding identified after initial screening), yielding 298 valid cases for analysis. The three groups comprised 99, 99, and 100 patients in the 1-h, 2-h, and 4-h groups, respectively (Figure 1). Baseline demographic and clinical characteristics were extracted from the electronic medical records and are presented in Table 1.

Comorbidities relevant to gastric transit were not prespecified variables in the original registry. Diabetes mellitus, Ehlers-Danlos syndrome, symptoms of gastroparesis, and objective gastric-emptying results were therefore not systematically abstracted, and their prevalence in the screened cohort cannot be reported reliably. Patients with pyloric obstruction or previous upper gastrointestinal surgery were excluded.

### Equipment, drugs, and reagents

Endoscopic examinations were performed using Olympus 290 and 260 electronic gastroenteroscopy systems and a Fujifilm 4450 electronic gastroenteroscopy system. The *H. pylori* breath test analyzer was manufactured by Anhui Yanghe Medical Company.

Pre-procedural mucosal preparation included oral administration of dimethicone powder (SFDA approval number H51023869; 2.5 g/bottle) and pronase (Beijing Tide Pharmaceutical Co., Ltd.; SFDA approval number H20110030; 20,000 units/box) 10–15 min before endoscopy for mucosal cleansing and defoaming. This interval is within the timing range shown to improve gastric mucosal visibility in controlled studies (41, 42). Sedation was achieved with intravenous propofol emulsion injection (Guangdong Jiabo Pharmaceutical Co., Ltd.; SFDA approval number H20051843; 10 mg/mL) or etomidate emulsion injection (Jiangsu Enhua Pharmaceutical Co., Ltd.; SFDA approval number H20020511). The choice of sedative agent was determined by the attending anesthesiologist on the basis of individual patient characteristics and was not a study variable.

The rapid diagnostic kit for *H. pylori* (urease method) was sourced from Shanghai Huitai Medical Technology Co., Ltd. (Shanghai Medical Device Registration Approval Number 20182400004). The *H. pylori* immunohistochemical (IHC) antibody reagent was obtained from Fuzhou Maixin Biotechnology Development Co., Ltd. (Fujian Medical Device Preparation No. 20180254). The gastric *H. pylori* special staining solution (methylene blue method) was supplied by Zhuhai Baso Biotechnology Co., Ltd. (batch number C240701; 20 mL/bottle). All biopsy specimens were fixed in 4% paraformaldehyde tissue fixative.

### Procedures

#### Urea [^14^C] breath test

As documented in the procedural records, the UBT was performed at the designated post-procedural interval (1, 2, or 4 h after completion of gastroscopy) according to the day-procedure unit's standard protocol and the manufacturer's instructions, consistent with consensus-based principles for testing active *H. pylori* infection (43). Each patient ingested one urea [^14^C] capsule with approximately 20 mL of cold water and then sat quietly for 25 min. A gas collection card was attached to a breathing tube; the patient breathed normally for 10 s and then exhaled smoothly and continuously through the tube for 2–5 min until the indicator changed from blue to white. The card was inserted into the detector. A result ≥100 disintegrations per minute (dpm) per mmol CO_2_ was classified as positive and < 100 dpm/mmol CO_2_ as negative. Trained technicians performed and interpreted all UBTs independently of the endoscopic findings.

#### Endoscopic examination

**Figure 2:**
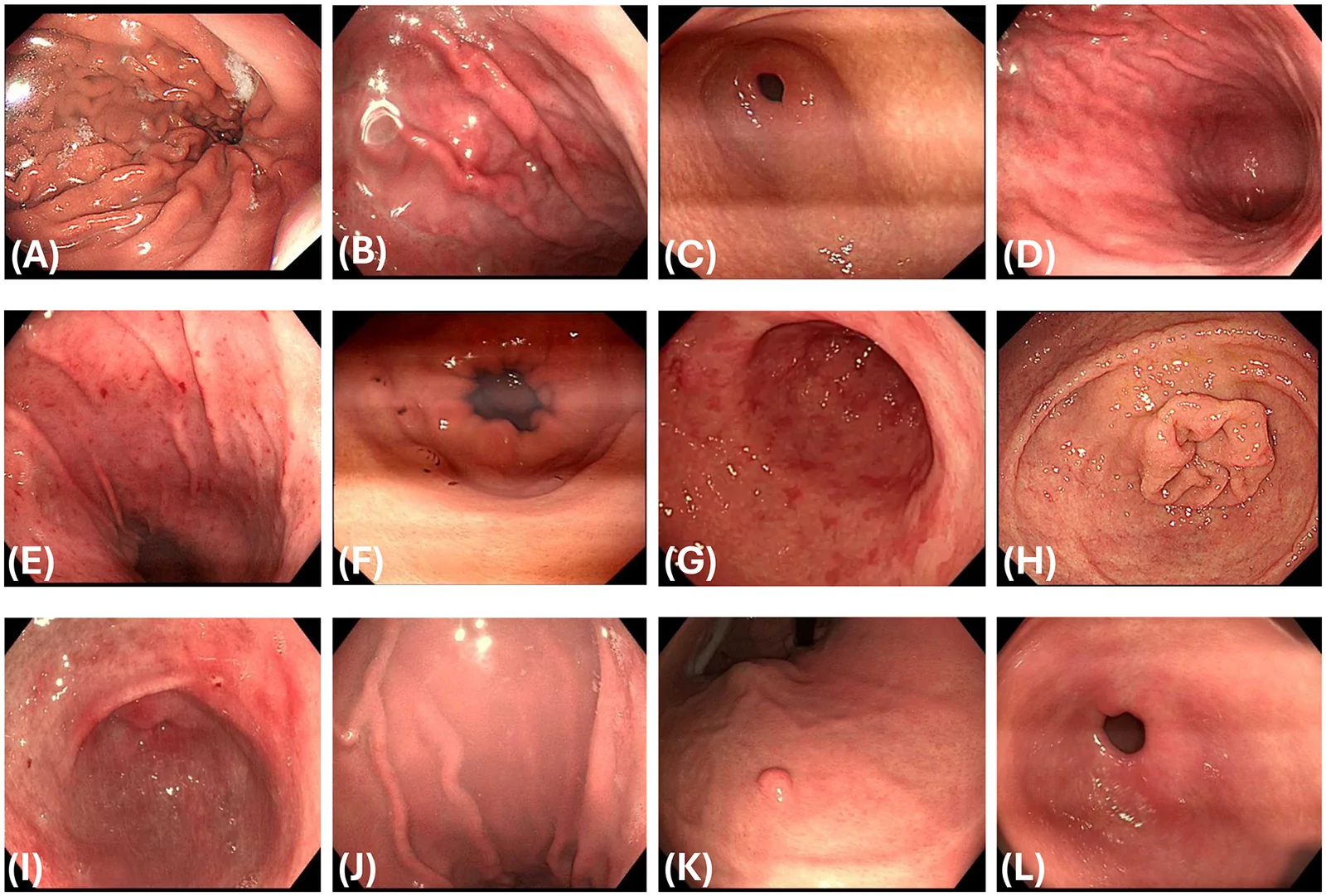
Representative white-light endoscopic findings used in the Kyoto Classification of Gastritis for assessment of *H. pylori* infection status. **(A)** Sticky mucus; **(B)** enlarged gastric folds; **(C)** nodularity; **(D)** diffuse redness; **(E)** spotty redness; **(F)** raised erosion; **(G)** map-like redness; **(H)** intestinal metaplasia; **(I)** depressed erosion; **(J)** loss of regular arrangement of collecting venules (RAC); **(K)** fundic gland polyp; **(L)** red streak. Sticky mucus, enlarged folds, nodularity, diffuse redness, and spotty redness are findings suggestive of current *H. pylori* infection. Map-like redness suggests previous infection, while intestinal metaplasia and loss of RAC indicate current or past infection. Fundic gland polyp and red streak are typically observed in *H. pylori*-negative mucosa. Raised erosion and depressed erosion are non-specific findings and should not be used alone to determine *H. pylori* infection status.

Patients fasted overnight (≥8 h) before the procedure. Under intravenous sedation, a gastroenterology specialist performed the examination according to standard endoscopic protocols, sequentially inspecting the esophagus, stomach, and duodenum. Gastric mucosal morphology was assessed under white-light endoscopy in accordance with the Kyoto global consensus report on *Helicobacter pylori* gastritis (20, 21), with systematic documentation of mucosal findings including nodularity, diffuse redness, raised erosion, punctate redness, xanthoma, hyperplastic polyps, intestinal metaplasia, mucosal atrophy, and chicken skin-like appearance, to assess *H. pylori* infection status. Representative endoscopic appearances are illustrated in Figure 2.

Biopsy sampling followed a standardized protocol informed by the updated Sydney approach to gastric mapping (27, 44). Two specimens were obtained from the posterior wall of the gastric antrum, 2–3 cm proximal to the pylorus, with one used for the rapid urease test (RUT) and the other for IHC and special staining; one specimen was taken from the posterior wall of the gastric body, 8 cm distal to the cardia, for pathological assessment of *H. pylori*; and additional specimens were collected from any abnormal lesion as clinically indicated. The total number of biopsies and the volume of irrigation fluid used during each procedure were recorded as procedural variables for subsequent analysis.

All endoscopic procedures were performed by four experienced gastroenterologists, each of whom had performed more than 1,000 endoscopic examinations and had been trained in the Kyoto classification system. For the purposes of this study, endoscopic assessment of *H. pylori* infection status was independently re-reviewed by two endoscopists through retrospective image review; disagreements were resolved by consensus with a third senior endoscopist with experience exceeding 10,000 procedures.

#### Pathological examination

All biopsy specimens were placed in individually labeled bottles containing 10% neutral buffered formalin and submitted to the pathology laboratory for routine processing. Histological assessment followed established gastric-biopsy principles, including evaluation of *H. pylori* and gastritis according to the updated Sydney framework (27, 44). Three complementary techniques were used for comprehensive *H. pylori* assessment.

Hematoxylin and eosin (H&E) staining. Paraffin-embedded tissue blocks were sectioned at 4 μm, mounted on adhesive-coated glass slides, and stained using a standardized H&E protocol. Sections underwent xylene deparaffinization, graded ethanol rehydration, hematoxylin staining, differentiation and bluing, eosin counterstaining, dehydration, clearing, and coverslip mounting, consistent with routine gastric-biopsy histology (27, 44).

**Figure 3:**
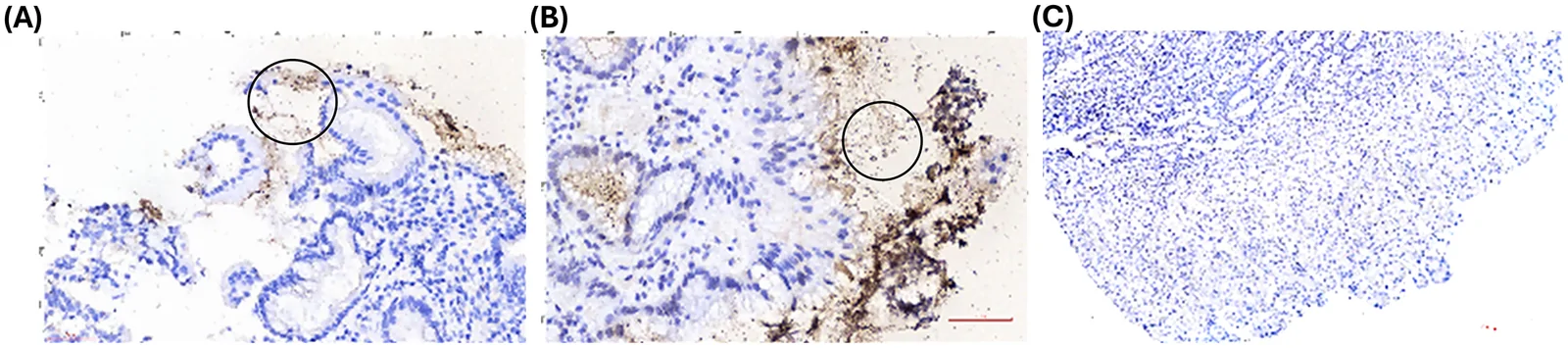
Immunohistochemical staining of gastric mucosa for *H. pylori*. **(A, B)** Positive immunohistochemical staining showing brown *H. pylori* organisms on the gastric mucosal surface or within gastric pits, as indicated by the black circles; original magnification, ×200. **(C)** Negative immunohistochemical staining, with no visible *H. pylori* organisms.

Immunohistochemical (IHC) staining for *H. pylori*. After deparaffinization and rehydration, sections underwent EDTA antigen retrieval (20 min), endogenous peroxidase blocking (10 min at room temperature), incubation with *anti-H. pylori* antibody (60 min at room temperature), reaction enhancer solution (15 min), and enzyme-labeled anti-mouse/rabbit IgG polymer (20 min), followed by 3,3′-diaminobenzidine chromogen development (1–10 min under microscopic monitoring), hematoxylin counterstaining, dehydration, clearing, and mounting. All steps included three phosphate-buffered saline washes of 5 min between reagent applications. The use of IHC to improve detection in low-density or morphologically atypical infection is supported by comparative pathology studies (33, 34). Representative positive and negative IHC results are shown in Figure 3.

**Figure 4:**
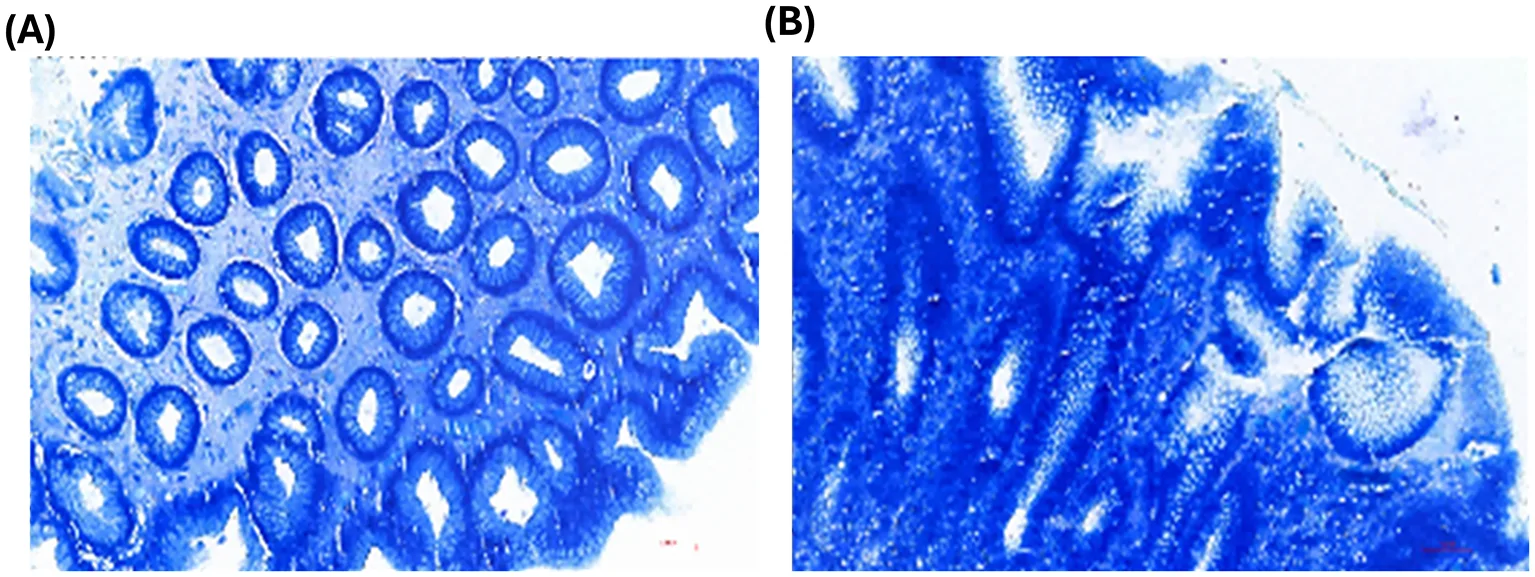
Methylene blue staining for *H. pylori* in gastric mucosal tissue. **(A)** Negative staining, with no visible blue or blue-purple curved bacilli. **(B)** Positive staining, showing blue/blue-purple curved bacilli consistent with *H. pylori* in the gastric pit or glandular area, as indicated by the black circle.

**Special staining (methylene blue method)**. Deparaffinized sections were stained with methylene blue solution for 5–10 min, rinsed with running water, differentiated briefly with 0.2% acetic acid, rinsed again, and treated with a weakly alkaline re-bluing solution, followed by graded ethanol dehydration, xylene clearing, and neutral gum mounting. *H. pylori* appeared as deep-blue or blue-purple short rods, arc-shaped or seagull-shaped organisms, characteristically clustered on the mucous layer of the gastric surface, in gastric pits, and in glandular lumina. Representative negative and positive methylene blue staining results are shown in Figures 4A, B, respectively.

#### Rapid urease test

In accordance with the manufacturer's instructions and established RUT methodology (10–12), the kit was equilibrated to room temperature (25 °C−35 °C) for 30 min before use. The dedicated antral biopsy specimen was inserted into the agar gel using pointed forceps. A color change from yellow to red with halo expansion within 5 min was recorded as positive; specimens without immediate color change were observed for an additional 30 min, with late color change recorded as weakly positive. Absence of color change after 30 min was recorded as negative.

### Diagnostic criteria

#### Reference standard for *H. pylori* Infection

To avoid incorporation bias, the reference standard was defined independently of the index test (UBT). A patient was classified as having current *H. pylori* infection if either of the following criteria was met: (1) positive IHC staining for *H. pylori* on gastric mucosal biopsy; or (2) in IHC-negative cases, concordant positive results on both the RUT and methylene blue special staining. This composite standard prioritizes direct histopathological evidence and requires dual concordance from independent biopsy-based methods when IHC is negative (28, 44–46). The approach was selected to maximize diagnostic certainty while limiting under-detection from reliance on a single biopsy-based technique, particularly when bacterial density is low.

#### Endoscopic interpretation criteria

Endoscopic signs of *H. pylori* infection were recorded according to the Kyoto global consensus report and the Kyoto gastritis classification score (20, 21). Documented features included regular arrangement of collecting venules (RAC), fundic gland polyps, raised redness, raised erosion, intestinal metaplasia, enlarged folds, chicken skin–like mucosa, depressed erosion, diffuse redness, map-like redness, multiple white flat protrusions, and mucosal atrophy (Figures 2A–L). Atrophy was classified using the Kimura–Takemoto system: closed types (C1, C2, C3) denoting atrophic borders confined within the cardia, and open types (O1, O2, O3) denoting borders extending beyond the cardia. In the Kyoto scoring system, no atrophy and C1 were scored as 0 points, C2–C3 as 1 point, and O1–O3 as 2 points.

#### Histopathological staging

All pathological sections were re-interpreted for the purposes of this study by two experienced pathologists who were blinded to clinical data, endoscopic findings, and UBT results. In cases of discordant interpretation, a third senior pathologist adjudicated the final classification. The histopathological staging of *H. pylori* invasion of the gastric mucosa (31) was classified across five progressive stages: Stage I (mucosal layer stage), characterized by *H. pylori* visible on epithelial cells; Stage II, encompassing mucosal cell degeneration (IIA) and mucosal erosion (IIB); Stage III, comprising diffuse acute and chronic inflammation (IIIA), compensatory hyperplasia (IIIB), ulceration (IIIC), and diffuse lymphocyte proliferation (IIID); Stage IV, representing glandular atrophy without (IVA) or with (IVB) intestinal metaplasia; and Stage V, denoting low-grade (VA) and high-grade (VB) intraepithelial neoplasia.

IHC staining density was graded as none (no *H. pylori* observed), mild (occasional bacteria or coverage of < 1/3 of specimen length), moderate (distribution over 1/3–2/3 of specimen length without continuity), or severe (clustered bacteria covering the full specimen length). Bacterial morphology was classified as spiral (typical helical, arc, or rod forms) or non-spiral (coccoid forms).

### Statistical analysis

Statistical analyses were performed using IBM SPSS Statistics for Windows, version 27.0 (IBM Corp., Armonk, NY, USA) (47). A two-sided *P* value < 0.05 was considered statistically significant. Categorical variables are presented as frequencies and percentages. Continuous variables are expressed as mean ± standard deviation for normally distributed data or median (interquartile range) for non-normally distributed data; normality was assessed using the Shapiro–Wilk test.

For comparisons of baseline characteristics among the three groups, one-way analysis of variance (ANOVA) was used for normally distributed continuous data with homogeneity of variance (verified by Levene's test), and the Kruskal–Wallis *H* test for data violating these assumptions. Categorical variables were compared using Pearson's chi-square test or, when any expected cell frequency was < 5, Fisher's exact test.

The diagnostic performance of the UBT at each time point was evaluated against the reference standard. Sensitivity, specificity, positive predictive value (PPV), and negative predictive value (NPV) were calculated with 95% confidence intervals derived using the Wilson score method (48). Agreement between the UBT and the reference standard was quantified using Cohen's kappa (κ), with 95% confidence intervals calculated from the asymptotic standard error. Kappa values were interpreted using the commonly cited Landis–Koch categories: < 0.20, poor; 0.21–0.40, fair; 0.41–0.60, moderate; 0.61–0.80, substantial; and 0.81–1.00, almost perfect agreement (49).

The association between histopathological stages and UBT results (true positive vs. false negative) within each time-point group was examined using the Fisher–Freeman–Halton exact test, selected because of small expected cell counts. The potential influence of endoscopic procedural factors (number of biopsies, irrigation volume) and mucosal background conditions (atrophy, intestinal metaplasia) on UBT diagnostic accuracy was explored using binary logistic regression, with UBT diagnostic status (correct vs. incorrect) as the dependent variable; odds ratios (ORs) with 95% CIs are reported. A supplementary analysis was conducted to assess the robustness of the primary findings using IHC positivity alone as the sole gold standard, representing the most stringent histopathological criterion for defining current infection. Because each post-gastroscopy time point represents a distinct clinical scenario rather than parallel tests of the same hypothesis, no formal correction for multiple comparisons was applied across time-point analyses; results are interpreted on the basis of the magnitude and directional consistency of diagnostic-performance estimates rather than on a single dichotomous significance threshold.

## Results

### Baseline characteristics and study population

Of the 300 patients identified during the case-finding period, 298 met all eligibility criteria and were included in the final analysis; 2 patients were excluded (1 for incomplete data and 1 for an undocumented history of upper gastrointestinal bleeding identified after initial screening), as detailed in the study flow diagram (Figure 1). The three post-gastroscopy time-point groups comprised 99, 99, and 100 patients in the 1-h, 2-h, and 4-h groups, respectively. Baseline demographic and clinical characteristics were well balanced across the three groups, with no statistically significant differences in sex, place of residence, dietary habits, smoking history, alcohol consumption, or ethnic distribution (all *P* > 0.05; Table 1).

### Endoscopic findings

Endoscopic examination of the 298 patients under white-light endoscopy revealed a broad spectrum of gastric mucosal abnormalities, summarized in Table 2 and illustrated in Figure 2. The most prevalent finding was enlarged folds, identified in 266 cases (89.3%), followed by diffuse redness in 162 cases (54.4%) and raised erosion in 124 cases (41.6%). With respect to mucosal atrophy, 86 patients (28.9%) showed no atrophy (C0), 165 (55.4%) had closed-type atrophy (C1–C3), and 46 (15.4%) had open-type atrophy (O1 or higher). Intestinal metaplasia was observed in 13 cases (4.4%). Additional findings included raised redness in 62 cases (20.8%), fundic gland polyps in 52 (17.4%), depressed erosion in 32 (10.7%), chicken skin–like mucosa in 9 (3.0%), regular arrangement of collecting venules (RAC) in 2 (0.7%), multiple white flat protrusions in 2 (0.7%), and map-like redness in 1 (0.3%). Representative endoscopic appearances of features associated with current infection (Figures 2A–E), previous infection (Figure 2G), current or previous infection (Figures 2H, J), *H. pylori*–negative status (Figures 2K, L), and non-specific findings (Figures 2F, I) are documented according to the Kyoto classification.

**Table 2:** Endoscopic findings of gastric mucosa (*N* = 298).

Finding	*n* (%)
RAC	2 (0.7%)
Fundic gland polyps	52 (17.4%)
Raised redness	62 (20.8%)
Raised erosion	124 (41.6%)
Atrophy C0 (none)	86 (28.9%)
Atrophy C1	72 (24.2%)
Atrophy C2	70 (23.5%)
Atrophy C3	23 (7.7%)
Atrophy O1 and above	46 (15.4%)
Intestinal metaplasia	13 (4.4%)
Mucosal fold swelling	266 (89.3%)
Chicken skin-like mucosa	9 (3.0%)
Depressed erosion	32 (10.7%)
Diffuse redness	162 (54.4%)
Map-like redness	1 (0.3%)
Multiple white flat protrusions	2 (0.7%)

Atrophy subtotals: C0 86 (28.9%) + C1 72 (24.2%) + C2 70 (23.5%) + C3 23 (7.7%) + O1 and above 46 (15.4%) = 297; one case was not classifiable. Closed-type atrophy (C1–C3) totals 165 (55.4%).

### Overall *H. pylori* infection status by detection method

On the basis of the independent composite reference standard, 140 of 298 patients (47.0%) were classified as having current *H. pylori* infection (Table 3). Among the individual diagnostic methods, the RUT demonstrated the highest positivity rate at 195 of 298 (65.4%), followed by the pooled UBT across all time points at 148 of 298 (49.7%), IHC staining at 140 of 298 (47.0%), and methylene blue special staining at 120 of 298 (40.3%). The discrepancy between the RUT positivity rate and the reference-standard prevalence reflects the known tendency of the RUT to yield a higher proportion of positive results, some of which may represent weakly positive reactions not corroborated by histopathological confirmation.

**Table 3:** Positivity rates of different diagnostic methods (*N* = 298).

Diagnostic method	Positive *n* (%)	Negative *n* (%)
Reference standard (IHC-based composite)	140 (47.0%)	158 (53.0%)
Rapid urease test (RUT)	195 (65.4%)	103 (34.6%)
Pathological special stain (Methylene Blue)	120 (40.3%)	178 (59.7%)
Urea [^14^C] breath test (all time points pooled)	148 (49.7%)	150 (50.3%)

### Agreement between UBT results and the reference standard at different post-gastroscopy time points

The cross-tabulation of UBT results against the reference standard at each post-gastroscopy time point, together with the corresponding Cohen's kappa coefficients, is presented in Table 4. Kappa analysis revealed substantial to almost-perfect agreement between the UBT and the reference standard across all three time points, with the highest concordance observed at the 2-h interval. Specifically, the kappa coefficient was 0.758 (95% CI: 0.673–0.843) at 1 h, 0.817 (95% CI: 0.745–0.889) at 2 h, and 0.704 (95% CI: 0.603–0.805) at 4 h (all *P* < 0.001; Table 4). These findings indicate that diagnostic agreement between the UBT and the histopathological reference standard was consistently substantial at all time points, with the 2-h post-gastroscopy interval achieving the highest degree of concordance.

**Table 4:** Cross-tabulation and agreement between [^14^C] UBT results and the reference standard.

Time Point	TP	FN	FP	TN	Kappa (95% CI)	*P*
1-h (*n* = 99)	43	5	7	44	0.758 (0.673–0.843)	< 0.001
2-h (*n* = 99)	40	7	2	50	0.817 (0.745–0.889)	< 0.001
4-h (*n* = 100)	43	2	13	42	0.704 (0.603–0.805)	< 0.001

TP, true positive (UBT+/Ref+); FN, false negative (UBT–/Ref+); FP, false positive (UBT+/Ref–); TN, true negative (UBT–/Ref–). Ref+/Ref–, positive or negative by the independent composite reference standard. 95% CI for kappa calculated via the asymptotic standard error.

### Diagnostic performance of the UBT at different time points

The sensitivity, specificity, PPV, and NPV of the UBT at each post-gastroscopy time point are presented in Table 5; the cross-tabulated counts on which these metrics are based are shown in Table 4. The diagnostic performance profile varied across time points, with each interval exhibiting distinct strengths suited to different clinical objectives.

**Table 5:** Diagnostic performance of the [^14^C] UBT at different time points.

Time point	Sensitivity (95% CI)	Specificity (95% CI)	PPV (95% CI)	NPV (95% CI)
1-h	89.6% (78.6–95.7)	86.3% (74.3–93.2)	86.0% (74.2–93.0)	89.8% (78.5–95.6)
2-h	85.1% (72.3–92.6)	96.2% (87.2–99.0)	95.2% (84.2–98.7)	87.7% (76.8–93.9)
4-h	95.6% (85.2–98.8)	76.4% (63.7–85.6)	76.8% (64.2–86.0)	95.5% (84.9–98.7)

PPV, positive predictive value; NPV, negative predictive value. 95% CIs calculated using the Wilson score method.

At the 1-h time point, the UBT demonstrated a sensitivity of 89.6% (95% CI: 78.6–95.7%) and a specificity of 86.3% (95% CI: 74.3–93.2%), yielding a PPV of 86.0% (95% CI: 74.2–93.0%) and an NPV of 89.8% (95% CI: 78.5–95.6%). The 2-h time point achieved the highest specificity at 96.2% (95% CI: 87.2–99.0%) and the highest PPV at 95.2% (95% CI: 84.2–98.7%), with a sensitivity of 85.1% (95% CI: 72.3–92.6%) and an NPV of 87.7% (95% CI: 76.8–93.9%). The 4-h time point yielded the highest sensitivity at 95.6% (95% CI: 85.2–98.8%) and the highest NPV at 95.5% (95% CI: 84.9–98.7%), though with a lower specificity of 76.4% (95% CI: 63.7–85.6%) and a PPV of 76.8% (95% CI: 64.2–86.0%). The wider 95% CI observed for specificity at the 4-h time point reflects both the lower point estimate (proportions farther from 1.0 are estimated with less precision) and the greater number of false-positive results in this group (Table 4).

Taken together, these results indicate that the 2-h post-gastroscopy interval provides the optimal balance of specificity and positive predictive value for precise clinical confirmation of *H. pylori* infection, whereas the 4-h interval is best suited for screening purposes in which maximizing sensitivity and negative predictive value is the priority.

### Reliability of endoscopic assessment of *H. pylori* infection status

Among the 298 patients, endoscopic assessment based on the Kyoto classification identified 140 cases (47.0%) as having current *H. pylori* infection and 158 cases as non-current infection (previous infection or negative). When compared with the histopathology-based reference standard, endoscopic assessment demonstrated substantial agreement, with a kappa coefficient of 0.758 (*P* < 0.001; Table 6). The concordance matrix revealed that endoscopy correctly identified 122 of 140 reference-positive cases and 140 of 158 reference-negative cases, yielding 18 false-positive and 18 false-negative endoscopic assessments. This corresponds to an endoscopic sensitivity of 87.1% (122/140) and a specificity of 88.6% (140/158) for identifying current *H. pylori* infection. These data affirm the utility of systematic white-light endoscopic evaluation according to the Kyoto classification as a moderately reliable adjunct for assessing *H. pylori* infection status, although histopathological confirmation remains the definitive diagnostic standard.

**Table 6:** Agreement between endoscopic assessment and reference standard for *H. pylori* infection status.

Endoscopic assessment	Reference standard (+)	Reference standard (–)	Total
Endoscopy: current infection	122	18	140
Endoscopy: non-current infection	18	140	158
**Total**	**140**	**158**	**298**

Kappa = 0.758 (*P* < 0.001). Endoscopic sensitivity = 122/140 = 87.1%; endoscopic specificity = 140/158 = 88.6%.

### Histopathological staging of true-positive and false-negative UBT cases

To investigate whether the severity of *H. pylori*–induced mucosal injury influenced UBT diagnostic accuracy, the distribution of histopathological stages among true-positive and false-negative UBT cases was analyzed at each time point (Table 7). A statistically significant difference in the distribution of mucosal injury stages between true-positive and false-negative cases was observed only at the 1-h time point (Fisher–Freeman–Halton exact test, *P* = 0.044). At this early interval, all five false-negative cases were concentrated in Stage III (diffuse inflammation and compensatory changes), whereas true-positive cases were distributed across Stages I through IV, with the majority in Stages II and III. Notably, no false-negative case at any time point was classified as Stage I, suggesting that when *H. pylori* colonization is confined to the superficial epithelium without inducing deeper inflammatory or degenerative changes, UBT performance is not compromised.

**Table 7:** Histopathological stage distribution among true-positive and false-negative UBT cases.

Time point	Stage	True positive (*n*)	False negative (*n*)	*P* value
1-h	I	4	0	0.044
	II	22	0	
	III	16	5	
	≥IV	1	0	
2-h	I	0	0	0.690
	II	18	4	
	III	18	3	
	≥IV	4	0	
4-h	I	2	0	1.000
	II	27	1	
	III	14	1	
	≥IV	0	0	

Fisher–Freeman–Halton exact test used due to small expected cell counts.

At the 2-h and 4-h time points, the histopathological stage distribution among false-negative cases (7 and 2 cases, respectively) did not differ significantly from that of true-positive cases (*P* = 0.690 and *P* = 1.000, respectively; Table 7). At 2 h, false-negative cases were distributed across Stages II (*n* = 4) and III (*n* = 3), and at 4 h, the two false-negative cases occurred in Stages II (*n* = 1) and III (*n* = 1). These findings suggest that the influence of mucosal injury severity on UBT accuracy is most pronounced at the 1-h interval and diminishes with increasing post-gastroscopy recovery time.

### Influence of procedural and mucosal factors on UBT diagnostic accuracy

Binary logistic regression was performed to explore whether endoscopic procedural factors or mucosal background conditions influenced UBT diagnostic accuracy at each time point, with UBT diagnostic correctness (correct vs. incorrect) as the dependent variable (Table 8). The factors examined were number of biopsy specimens (3, 4, or 5), irrigation volume (< 60 ml, 60–90 ml, or >90 ml), presence and degree of mucosal atrophy (none, closed-type, or open-type), and presence of intestinal metaplasia.

**Table 8:** Influence of procedural and mucosal factors on UBT diagnostic accuracy.

Factor	Category	Code	1 h (*n*)	*P*	2 h (*n*)	*P*	4 h (*n*)	*P*
Number of biopsies	3	1	28	0.843	27	0.624	29	0.173
	4	2	62		66		62	
	5	3	9		6		9	
Water flush volume	< 60 ml	1	1	0.917	0	0.998	0	0.212
	60–90 ml	2	53		68		58	
	>90 ml	3	45		31		42	
Atrophy	None	0	26	0.998	31	0.126	29	0.743
	Closed-type	1	60		53		52	
	Open-type	2	12		15		19	
Intestinal metaplasia	Yes	0	5	0.093	4	0.998	4	0.242
	No	1	94		95		96	
Diagnostic status	Correct	1	87		90		85	
	Incorrect	0	12		9		15	

Binary logistic regression with UBT diagnostic correctness (correct = 1; incorrect = 0) as the dependent variable. Counts within each factor sum to the group total (1 h, *n* = 99; 2 h, *n* = 99; 4 h, *n* = 100).

None of the examined factors showed a statistically significant association with UBT diagnostic accuracy at any of the three time points. For number of biopsy specimens, *P* values were 0.843, 0.624, and 0.173 at 1, 2, and 4 h, respectively. Irrigation volume similarly showed no significant effect (*P* = 0.917, 0.998, and 0.212 at 1, 2, and 4 h). Mucosal atrophy (*P* = 0.998, 0.126, and 0.743) and intestinal metaplasia (*P* = 0.093, 0.998, and 0.242) were also non-significant across all time points (Table 8). However, the effect of intestinal metaplasia on UBT accuracy could not be reliably assessed in this analysis, because only 13 patients (4.4%) had histologically confirmed intestinal metaplasia (5, 4, and 4 in the 1-h, 2-h, and 4-h groups, respectively), yielding insufficient statistical power for this variable and contributing to the computational instability reflected in the *P* = 0.998 at 2 h. These null findings suggest that, within the ranges observed in this study, routine procedural variables associated with standard diagnostic gastroscopy do not materially compromise the diagnostic reliability of the post-gastroscopy UBT.

### Sensitivity analysis

To assess the robustness of the primary findings, a supplementary analysis was performed using positive IHC staining alone as the sole gold standard for defining current *H. pylori* infection, representing the most stringent histopathological criterion. Under this criterion, 140 patients (47.0%) were classified as infected, a number identical to that under the composite reference standard, indicating that all reference-positive cases in this dataset were confirmed by IHC positivity and that the dual-concordance pathway (IHC-negative with both RUT and special stain positive) did not reclassify any additional patients (Supplementary Table S1).

The recalculated diagnostic-performance metrics at each time point were identical to those of the primary analysis: the 1-h time point showed a sensitivity of 89.6% and a specificity of 86.3%; the 2-h time point showed a sensitivity of 85.1% and a specificity of 96.2%; and the 4-h time point showed a sensitivity of 95.6% and a specificity of 76.4% (Supplementary Table S1). The consistency of these results across both reference-standard definitions confirms that the principal conclusions regarding optimal post-gastroscopy UBT timing are robust and are not dependent on the specific formulation of the diagnostic reference criterion.

## Discussion

This study addresses a practical question that is not resolved by standard *H. pylori* testing guidance: whether a capsule-based urea [^14^C] breath test remains diagnostically useful when performed during recovery after gastroscopy. Using a reference standard defined independently of the UBT (28, 44–46), we found clinically informative agreement at all three intervals, with different timing windows emphasizing different diagnostic properties.

The reference-standard prevalence was 47.0%, which is compatible with the substantial burden of *H. pylori* reported in China and other high-prevalence settings (3). Because this was a symptomatic clinical cohort rather than a population survey, the observed prevalence should not be interpreted as a regional population estimate. The absence of statistically significant differences by sex, residence, dietary preference, smoking, alcohol use, or ethnicity may reflect limited power for epidemiological subgroup analyses rather than evidence that these factors are unrelated to infection.

Across methods, RUT positivity exceeded UBT and IHC positivity. This pattern supports the decision not to use RUT alone as the reference standard because weak color reactions can be affected by sampling, bacterial density, and non-*H. pylori* urease activity. UBT remains a recommended non-invasive test, while systematic Kyoto-based white-light endoscopy provides complementary information but cannot replace tissue-based or validated non-invasive confirmation (5, 7, 43).

At 2 h, UBT had the highest kappa, specificity, and PPV; at 4 h, it had the highest sensitivity and NPV. These findings are clinically relevant timing-specific profiles rather than proof of statistical superiority, because the groups were independent and the study was not designed as a head-to-head non-inferiority trial. Prior work in patients tested immediately after emergency endoscopy for peptic ulcer bleeding reported reduced specificity for an early UBT, suggesting that very early post-procedural testing and acute bleeding can introduce additional variability (50). More broadly, meta-analytic and comparative evidence confirms high sensitivity for [^14^C] UBT but also shows that specificity depends on assay platform, cut-off selection, and the handling of borderline results (51, 52). Our findings extend that literature by anchoring three routine post-gastroscopy intervals to histopathology in non-bleeding adults.

The concentration of 1-h false-negative results in Stage III mucosal injury is hypothesis-generating. Active inflammation, edema, altered mucus, focal bacterial distribution, biopsy-related disruption, or transient dilution could reduce the amount or distribution of urease activity available to the ingested substrate shortly after endoscopy. However, only five false-negative cases occurred at 1 h, and no mechanistic measurements were obtained. The finding should therefore not be interpreted as proof that Stage III disease causes false negativity; it identifies a subgroup for prospective confirmation using paired pre- and post-gastroscopy UBT and quantitative bacterial-density measures.

All patients received pronase and dimethicone 10–15 min before endoscopy. Controlled studies support premedication within this interval for improving gastric mucosal visibility (41, 42), so the protocol was consistent with validated endoscopic practice. Delayed gastric emptying can occur in diabetes and has also been reported in hypermobile Ehlers-Danlos syndrome (53, 54). A meta-analysis has also evaluated the diagnostic performance of UBT, RUT, and histology after partial gastrectomy (55); patients with previous upper gastrointestinal surgery were excluded from the present cohort. The original registry did not systematically capture these diagnoses, gastroparesis symptoms, motility-altering diabetes medications, or gastric-emptying studies; consequently, we cannot determine how many participants had delayed transit or whether it modified UBT performance. We found no evidence that empirically administering the defoaming and mucolytic preparation earlier would prevent UBT bias, because the preparation is intended to improve endoscopic visibility rather than standardize post-procedural isotope distribution. In patients with known or suspected gastroparesis, a separately scheduled UBT after full recovery and an appropriate fasting period is a reasonable precaution until prospective data are available.

The procedural and mucosal factors examined, including biopsy number, irrigation volume, mucosal atrophy, and intestinal metaplasia, showed no statistically significant association with UBT diagnostic accuracy at any time point. These analyses were exploratory and should not be interpreted as demonstrating equivalence or absence of effect. Irrigation volume was recorded in broad categories, total procedure time and insufflation were unavailable, and only 13 patients had intestinal metaplasia. Sparse data likely contributed to unstable logistic estimates and near-unity *P* values. Prospective studies should record procedural exposure continuously and oversample clinically relevant mucosal subgroups.

This study has several limitations. First, it was conducted at a single center with a moderate sample size. Although each timing group exceeded the retrospective precision benchmarks, a larger multicenter cohort would improve external validity and permit more stable subgroup analyses. The 1-, 2-, and 4-h intervals were determined by the unit's existing scheduling structure; a 3-h group was not available. Second, the retrospective timing groups were defined by clinical scheduling rather than random allocation, so residual confounding cannot be excluded despite balanced measured characteristics. Third, the reference standard was independent of the UBT but necessarily depended on biopsies obtained during the endoscopic procedure under investigation. Fourth, procedural exposures were incompletely quantified, and diabetes, Ehlers-Danlos syndrome, prokinetic or motility-altering diabetes therapy, symptoms of gastroparesis, and objective gastric-emptying data were not systematically available. Delayed gastric transit therefore could not be evaluated. Fifth, the cohort came from one region of Southwest China and may differ from other populations in prevalence, bacterial strains, and host factors. Finally, no pre-gastroscopy UBT comparator was available; a paired prospective design would provide the strongest direct assessment of procedure-related change.

## Conclusion

In this cohort, same-day urea [^14^C] breath testing after gastroscopy produced clinically informative results across the evaluated intervals. The 2-h interval favored specificity and positive predictive value, whereas the 4-h interval favored sensitivity and negative predictive value. These estimates support pragmatic scheduling according to clinical purpose but do not establish formal superiority between independently scheduled groups. The association between Stage III mucosal injury and false-negative results at 1 h requires prospective confirmation. Future paired and multicenter studies should also evaluate delayed gastric transit and more precisely measured procedural exposures.

## Data Availability

The original contributions presented in the study are included in the article/Supplementary material, further inquiries can be directed to the corresponding author.
