# Supplementary material for: Optimal post-gastroscopy timing of the urea [14C] breath test for Helicobacter pylori diagnosis: a histopathology-anchored diagnostic accuracy study
Source: Front Med (Lausanne). 2026 Aug 25;13:1935940. doi: 10.3389/fmed.2026.1935940 (PMC13549848; doi:10.3389/fmed.2026.1935940)
Supplement: Supplementary file 1 [file Supplementary_file_1.docx]

**Supplementary Table S1. Diagnostic Performance of the Urea [14C] Breath Test at Different Time Points Using IHC Positivity Alone as the Gold Standard.**

| **Time Point** | **UBT Result** | **IHC+** | **IHC−** | **Total** |
| --- | --- | --- | --- | --- |
| 1-hour (n=99) | Positive | 43 | 7 | 50 |
|  | Negative | 5 | 44 | 49 |
| 2-hour (n=99) | Positive | 40 | 2 | 42 |
|  | Negative | 7 | 50 | 57 |
| 4-hour (n=100) | Positive | 43 | 13 | 56 |
|  | Negative | 2 | 42 | 44 |
| Overall (N=298) | Positive | 126 | 22 | 148 |
|  | Negative | 14 | 136 | 150 |

| **Time Point** | **Sensitivity (95% CI)** | **Specificity (95% CI)** | **PPV (95% CI)** | **NPV (95% CI)** | **Kappa (95% CI)** |
| --- | --- | --- | --- | --- | --- |
| 1-hour | 89.6% (78.6–95.7) | 86.3% (74.3–93.2) | 86.0% (74.2–93.0) | 89.8% (78.5–95.6) | 0.758 (0.673–0.843) |
| 2-hour | 85.1% (72.3–92.6) | 96.2% (87.2–99.0) | 95.2% (84.2–98.7) | 87.7% (76.8–93.9) | 0.817 (0.745–0.889) |
| 4-hour | 95.6% (85.2–98.8) | 76.4% (63.7–85.6) | 76.8% (64.2–86.0) | 95.5% (84.9–98.7) | 0.704 (0.603–0.805) |
| Overall | 90.0% (84.0–94.0) | 86.1% (79.9–90.7) | 85.1% (78.7–89.9) | 90.7% (85.0–94.4) | 0.758 (0.703–0.813) |

*Note:* Performance metrics under this strictest reference are identical to those under the composite reference standard reported in the main analysis (Table 5), confirming the robustness of the primary findings.
